# Lyophilized Hybrid Nanostructured Lipid Carriers to Enhance the Cellular Uptake of Verapamil: Statistical Optimization and In Vitro Evaluation

**DOI:** 10.1186/s11671-018-2744-6

**Published:** 2018-10-15

**Authors:** Arshad Ali Khan, Ibrahim M. Abdulbaqi, Reem Abou Assi, Vikneswaran Murugaiyah, Yusrida Darwis

**Affiliations:** 1 School of Pharmaceutical Sciences, Universiti Sains Malaysia, 11800 Minden, Penang Malaysia; 2 Faculty of Engineering Technology, Universiti Malaysia Pahang, Kuantan, Pahang Malaysia

**Keywords:** Verapamil hydrochloride, Dextran sulfate sodium, Nanostructured lipid carriers, Factorial design, Caco-2 cell line, Cellular uptake

## Abstract

Verapamil is a calcium channel blocker and highly effective in the treatment of hypertension, angina pectoris, and other diseases. However, the drug has a low bioavailability of 20 to 35% due to the first pass effect. The main objective of this study was to develop hybrid verapamil-dextran nanostructured lipid carriers (HVD-NLCs) in an attempt to increase verapamil cellular uptake. The formulations were successfully prepared by a high-shear homogenization method and statistically optimized using 2^4^ full factorial design. The HVD-NLCs formulations were freeze-dried using trehalose as a cryoprotectant. The results showed that the optimized formula (VER-9) possessed a particle size (PS), polydispersity index (PDI), and the percentage of entrapment efficiency (%EE) of 192.29 ± 2.98, 0.553 ± 0.075, and 93.26 ± 2.66%, respectively. The incorporation of dextran sulfate in the formulation had prolonged the release of verapamil (~ 85% in 48 h) in the simulated gastric fluid (pH 1.2) and simulated intestinal fluid (pH 6.8). The differential scanning calorimetry analysis showed no chemical interaction between verapamil and the excipients in the formulation. While wide-angle X-ray scattering studies demonstrated the drug in the amorphous form after the incorporation in the NLCs. The transmission electron microscopy and scanning electron microscopy images revealed that the nanoparticles had spherical shape. The cellular uptake study using Caco-2 cell line showed a higher verapamil uptake from HVD-NLCs as compared to verapamil solution and verapamil-dextran complex. The optimized formulation (VER-9) stored in the refrigerated condition (5 °C ± 3 °C) was stable for 6 months. In conclusion, the HVD-NLCs were potential carriers for verapamil as they significantly enhanced the cellular uptake of the drug.

## Background

Verapamil is an L-type calcium channel blocking agent of phenylalkylamine class and an antagonist of the α-adrenergic receptor. It is extensively used for the treatment of hypertension, supraventricular tachyarrhythmia, angina pectoris, and cluster headaches. According to the biopharmaceutics classification system, verapamil is classified as class I drug. The drugs under this class are known to have good absorption via intestinal membrane (≤ 90%) following oral administration. However, only 20 to 35% of the verapamil oral dose is passed to the blood circulation due to rapid first pass metabolism through portal circulation [1]. Therefore, it is important to develop suitable carriers that can enhance verapamil cellular uptake and possibly improve its bioavailability.

The second generation, lipid-based nanoformulations such as nanostructured lipid carriers (NLCs) exhibited a great potential for use in the delivery of therapeutic agents [2, 3]. The NLCs have higher chemical and physical stability in comparison to the solid lipid nanoparticles (SLNs), and also they have controlled release properties. Moreover, NLCs exhibit high loading capacity for drug molecules by forming a fewer ordered crystal lattice of lipid matrix, that could minimize the expulsion of the drug during storage. NLCs are prepared from lipids, which accelerate the chylomicron formation inside the intestines and facilitate the absorption of NLCs that may increase the bioavailability of drugs [4]. Therefore, the present study developed hybrid verapamil-dextran nanostructured lipid carriers (HVD-NLCs) to improve the cellular uptake of the drug that may lead to increase its bioavailability.

**Fig. 1:**
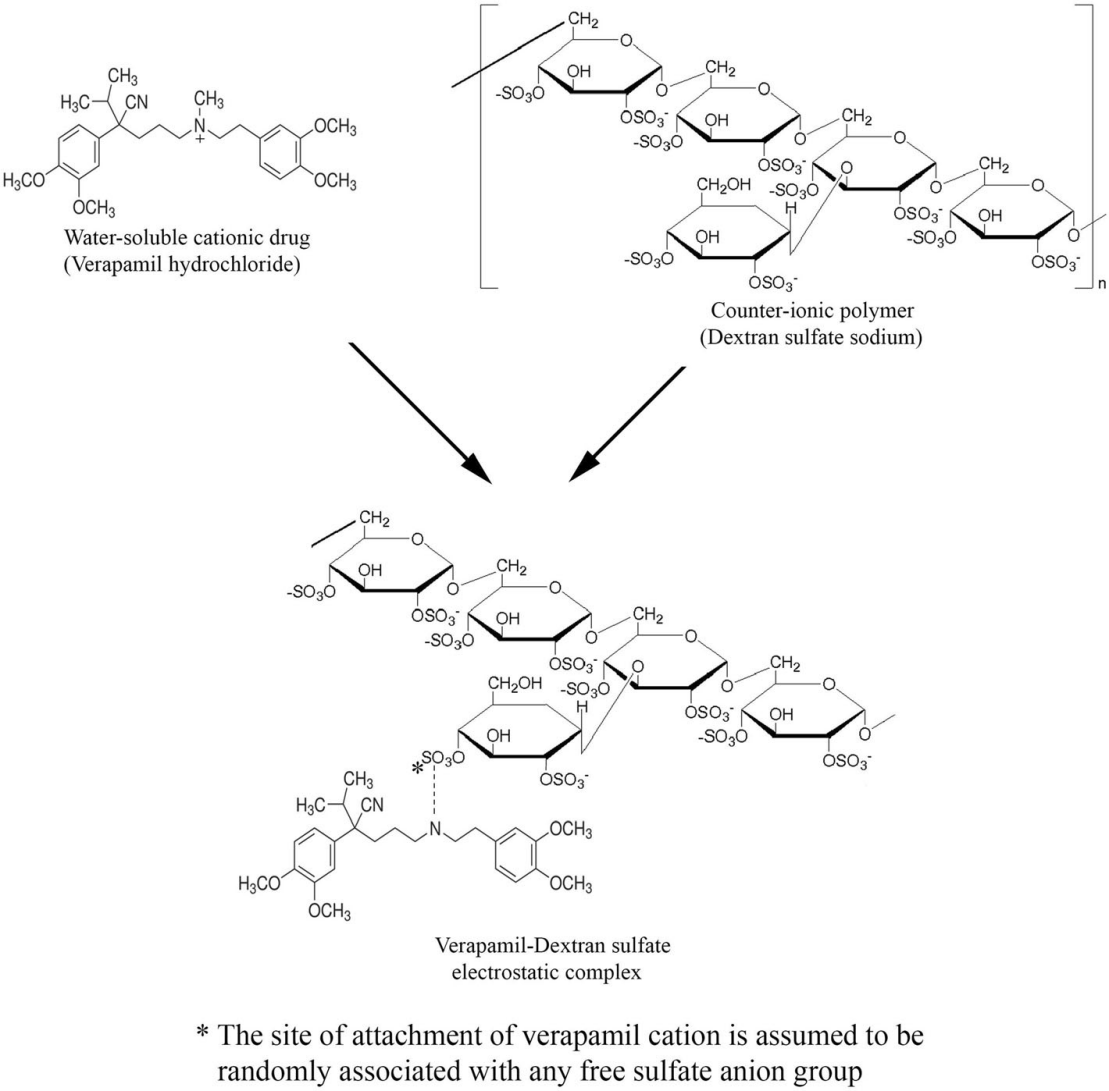
Formation of electrostatic complex of water-soluble cationic drug verapamil hydrochloride and counterion polymer dextran sulfate sodium

Most of the lipophilic drugs can easily incorporate into the NLCs, but it is quite difficult to entrap hydrophilic drugs like verapamil in a lipid-based nanoformulation. Thus, in the present study, hybrid NLCs using a counterion dextran sulfate sodium were prepared for enhancing the encapsulation of verapamil and prolonging its release from the NLCs (Fig. 1). The HVD-NLCs formulations were prepared by the hot and high-shear homogenization method and optimized statistically using a 2^4^ full factorial design. Two types of lipids were used in the study, the solid lipid, Compritol 888 ATO® and the liquid lipid oleic acid. The prepared HVD-NLCs were characterized for their particle size (PS), zeta potential (ZP), polydispersity index (PDI), and the percent of drug entrapment efficiency (%EE). The optimized HVD-NLCs were lyophilized using trehalose as a cryoprotectant. The in vitro release profiles were conducted in alkaline and acidic environments. The in vitro cellular uptake study of verapamil from the selected HVD-NLCs was performed using Caco-2 cell lines. The stability study of the optimized formulation was conducted for 6 months at three different conditions (5 °C ± 3 °C, 25 °C ± 2 °C/60% RH ± 5% RH, and 40 °C ± 2 °C/75% RH ± 5% RH).

## Methods

### Material

Verapamil HCl was purchased from the Wenzhou pharmaceutical factory (Wenzhou, China). Compritol 888 ATO® (Glycerol dibehenate EP–Glyceryl behenate NF) was obtained from Gattefosse (Saint-Priest, France). Oleic acid was procured from R & M chemicals (Essex, UK). Tween 80® (Polysorbate 80) was purchased from Euro chemo-pharma Sdn. Bhd. (Penang, Malaysia). Poloxamer 188 (Pluronic® F-68) was procured from Molekula (Dorset, UK). Trehalose, Sephadex® G-25, and fetal bovine serum (FBS) were obtained from Sigma-Aldrich (St. Louis, MO, USA). The Caco-2 cell line was obtained from ATCC (Virginia, USA). Penicillin-Streptomycin solution 100 X was obtained from Biowest (Nuaillé, France). Trypsin 0.25% and Dulbecco’s modified Eagles medium (DMEM) were procured from GE healthcare life sciences, Hyclone laboratories (Utah, USA). Passive Lysis Buffer, 5X was purchased from Promega (Wisconsin, USA).

### Methods

#### Preparation of HVD-NLCs

HVD-NLCs were prepared by the hot high-shear homogenization method. The lipid phase consisted of Compritol ATO 888® and oleic acid was melted by heating at 85 °C (10 °C above the melting point of solid lipid). The required amount of verapamil was weighed and dissolved in the aqueous phase containing a mixture of Tween 80® and poloxamer 188 at a ratio of 1:1 *w*/*w*. The aqueous phase was heated to the same temperature as lipid phase. The aqueous phase was poured into the lipid phase and homogenized using a digital T25 Ultra-Turrax® homogenizer, IKA® (Staufen, Germany) at 24,000 rpm, 85 °C. Subsequently, the dextran sulfate aqueous solution (1:1 ratio to verapamil) was added slowly into the mixture during the homogenization process. The prepared HVD-NLCs were left to cool at room temperature (25 ± 2 °C).

#### Statistical Optimization of the Formulation Parameters Using 2^4^ Full Factorial Design and Desirability Function Approaches

A full factorial design with two levels and four variables was performed to optimize and evaluate the effect of formulation independent variables (factors) on the characteristics (dependent variables or responses) (Table 1). The true high and low levels for each independent variable were selected based on the preliminary studies.

**Table 1:** Description of 2^4^ full factorial design

Factors (independent variables)	Low level	High level
A: Homogenization time	20.00 min	30.00 min
B: Solid lipid (Compritol 888 ATO®)	270.00 mg	285.00 mg
C: Liquid lipid (oleic acid)	15.00 mg	30.00 mg
D: Surfactants (Poloxamer 188: Tween 80®, 1:1)	200.00 mg	300.00 mg
Responses (dependent variables)	Constraints	
PS (nm)	Minimize	
PDI	Minimize	
ZP (mV)	Maximize	
EE (%)	Maximize	

*PS* particle size, *PDI* polydispersity index, *ZP* zeta potential, *EE* entrapment efficiency

The experimental responses obtained from the dependent variables were the results of the individual and combined effects of the four independent variables. This two-level experimental design provides sufficient data to fit the following polynomial equation.

1 $$ X={\upbeta}_0+{\upbeta}_1A+{\upbeta}_2B+{\upbeta}_3C+\upbeta 4D+{\upbeta}_{12} AB+{\upbeta}_{13}\mathrm{AC}+{\upbeta}_{14}\ \mathrm{AD}+{\upbeta}_{23}\mathrm{BC}+{\upbeta}_{24}\mathrm{BD}+{\upbeta}_{34}\mathrm{CD} $$

Where *X* is a dependent variable; β_0_ is an intercept; β_1_, β_2_, β_3_, and β_4_ are the coefficients of the independent variables *A*, *B*, *C*, and *D* respectively. While β_12_, β_13_, β_14_, β_23_, β_24_, and β_34_ are the respective interactions (i.e., AB, AC, AD, BC, BD, and CD). The experimental results were analyzed by a Design-Expert® software version 6.0.10 (Stat-Ease, Inc., Minneapolis, MN, USA) followed by analysis of variance (ANOVA) to determine the significance of factors and their interactions. The statistical analysis was considered significant when the *p* values were < 0.05.

The final formulations were selected on the basis of the desirability function approach. The desirability function combines all the responses into one variable to predict the optimum levels of the studied factors. Therefore, the desirability was determined to select the optimized formulations with the lowest particle size (PS) and polydispersity index (PDI) and the highest zeta potential (ZP) and percent entrapment efficiency (%EE) values. The desirability function converts all responses into a common scale (0, 1) and combines them by geometric mean for the optimization of overall metric. The desirability value 1 denotes an acceptable value (most desired value) for the responses, while desirability value 0 indicates an unacceptable value.

#### Measurements of Particle Size, Polydispersity Index, and Zeta Potential

The PS and PDI of the prepared nanoparticles were measured using a photon correlation spectrometer (Zetasizer 1000HS/3000HS, Malvern Instrument, Malvern, UK). The ZP was determined using a zeta potential analyzer (Zetasizer nano series, Nano Z-red badge, Malvern Instrument, Malvern, UK). All samples were diluted using filtered distilled water (0.45 μm Nylon filter) and measurements were done in triplicate manner.

#### Determination of Percent Entrapment Efficiency

The prepared sample containing free verapamil-dextran complex and HVD-NLCs were separated by mini-column centrifugation method using Sephadex® G25. A volume of 1 mL of the sample was placed on the top of the column, and then centrifuged for 2 min at 2000 rpm. The eluent that contained HVD-NLCs was collected and lyophilized using a freeze dryer (Labconco, Free Zone Freeze Dry Systems, Kansas City, USA).

The lyophilized HVD-NLCs (10 mg) was dissolved in chloroform and evaporated under nitrogen gas at 40 °C. The dried sample was then dissolved in 1 mL distilled water, vortexed for 2 min, and centrifuged (Eppendorf, Germany) at 12,000 rpm for 5 min. The supernatant was collected and the amount of verapamil was determined by using a validated high-performance liquid chromatography (HPLC) method. The %EE was calculated using the following equation:

2 $$ \%\mathrm{EE}=\frac{\mathrm{Weight}\ \mathrm{of}\ \mathrm{encapsulated}\ \mathrm{verapamil}}{\mathrm{Initial}\ \mathrm{weight}\ \mathrm{of}\ \mathrm{verapamil}}\kern0.5em \times \kern0.75em 100 $$

The HPLC analysis for quantification of verapamil was performed using Shimadzu chromatographic system fitted with LC 20AD delivery pump (Kyoto, Japan) and Phenomenex C18 column (250 × 4.6 mm). The mobile phase consisted of 20 mM ammonium acetate and acetonitrile at a ratio of 40:60 (*v*/*v*). The pH of the mobile phase was adjusted to pH 6.5 with glacial acetic acid. The injection volume was 20 μL and detection wavelength for verapamil was set at 278 nm and the flow rate was fixed at 1.5 mL/min.

#### Lyophilization Study

The lyophilization was done at − 40 °C for 24 h using a Labconco, FreeZone Freeze Dry Systems (Kansas City, USA). Five types of commonly used sugars, including mannitol, fructose, sucrose, lactose, and trehalose at lipid:cryoprotectant ratio of 1:1 were screened, in order to select a suitable cryoprotectant for the freeze-drying of the HVD-NLCs. The cryoprotectant which produced lowest mean PS and PDI of the lyophilized formulation after reconstitution in distilled water was selected for further study.

In the next study, the HVD-NLCs formulation was mixed with the selected cryoprotectant by using two methods. In the first method, the cryoprotectant was added after preparation of HVD-NLCs formulation, and it was named as “after homogenization process.” While in the second method, the cryoprotectant was added during the preparation of HVD-NLCs formulation, and it was named as “during homogenization process.” In the first method, several lipids and cryoprotectant ratios of 1:1, 1:2, 1:4, 1:6, and 1:8 were prepared and lyophilized. The lyophilized HVD-NLCs formulations were re-dispersed in the filtered, distilled water (0.45 μm Nylon filter), and the samples were evaluated for mean PS, PDI, and %EE. The lipid:cryoprotectant ratios that produced the lowest PS and PDI and highest %EE were selected for further studies in the second mixing method.

#### In Vitro Release Study

The in vitro release study of verapamil from NLCs was carried out using a dialysis bag placed in a simulated intestinal fluid (pH 6.8) or simulated gastric fluid (pH 1.2) medium. In this study, 2 mL of HVD-NLCs dispersion was filled into the dialysis bag (12,000 Da MW cutoff). The bag was sealed and then immersed in 150 mL of preheated release medium. The release study was performed on a hot plate magnetic stirrer set at 100 rpm stirring speed and 37 °C. At scheduled time intervals of 0.08, 0.25, 0.5, 1, 2, 4, 6, 8, 10, 12, 24, 48, and 72 h, 1 mL of sample was withdrawn and replaced with the same volume of fresh medium. The released verapamil concentration was determined by the HPLC.

#### Differential Scanning Calorimetry

The differential scanning calorimetry (DSC) analysis was carried out using a Perkin-Elmer Pyris 6 DSC (Perkin-Elmer, Beaconsfield, UK). The samples were weighed (5–7 mg) into an aluminum pan and sealed using a standard sample pan crimper press (Perkin-Elmer, Beaconsfield, UK). DSC curves were recorded at the rate of 10 °C/min from 0 to 260 °C.

#### Wide-Angle X-Ray Scattering

The wide-angle X-ray scattering (WAXS) θ/2θ analysis was performed at room temperature using a PANalytical X’Pert PRO MRD PW3040 (Almelo, Netherlands) applying Cu Kα radiation. The samples were run at the temperature range of 2–60 °C with a scanning rate of 5 °C/min.

#### Transmission Electron Microscope and Scanning Electron Microscope

The morphology (i.e., shape and size) of HVD-NLCs was observed by a transmission electron microscope (TEM) using (Philips CM12, Eindhoven, Netherlands). A drop of diluted HVD-NLCs dispersion was placed on a 400 mesh copper grid followed by a negative staining with 2% phosphotungstic acid. The prepared sample was air dried before the TEM examination.

The morphology of lyophilized HVD-NLCs was observed using a scanning electron microscope (SEM) (Leo Supra 50 VP field Emission SEM, Oberkochen, Germany). The lyophilized HVD-NLCs sample was fixed on an aluminum stub then coated with gold (10 nm thickness) in a sputtering device at 15 mA for 15 min. The sample was visualized using an Oxford INCA energy dispersive x-ray microanalysis system with accelerating voltage of 10 kV.

#### Cellular Uptake of HVD-NLCs

The in vitro cellular uptake study of verapamil from HVD-NLCs formulation was investigated using a Caco-2 cell line. The cells were cultured in DMEM growth media supplemented with 1% penicillin-streptomycin solution and 10% FBS in T-25 tissue culture flask. Then, the Caco-2 cells were collected and seeded in 48-well plates, at 60,000 cell density per well with DMEM complete media until the cells became confluent and formed a monolayer on the bottom of the well plate. Several solutions containing the same concentration of verapamil were prepared by diluting HVD-NLCs, verapamil solution, and verapamil-dextran complex with DMEM media only. Verapamil solution and verapamil-dextran complex were used as controls. Subsequently, the DMEM complete media of Caco-2 cells monolayer in 48-well plates were exchanged with different dilutions of HVD-NLCs, verapamil solution, and verapamil-dextran complex and incubated at 37 °C for 6 h. After completing the incubation period, the HVD-NLCs, verapamil solution, and verapamil-dextran complex samples were removed from the wells. The residue of the test samples and dead cells were removed from the monolayers by washing three times with phosphate-buffered saline (PBS). The cells of monolayer were lysed by adding passive lysis buffer (200 μL) to each sample well. Samples were pipetted out and centrifuged at 12,000 rpm for 10 min to extract the verapamil from the cells. The supernatants were collected and verapamil concentration was quantified using the HPLC.

#### Short-Term Stability Study

The short-term stability study of optimized HVD-NLCs formulation was performed according to the international council for harmonization of technical requirements for pharmaceuticals for human (ICH) guidelines Q1A (R2). The freshly prepared lyophilized HVD-NLCs formulation (VER-9) was placed at three different conditions (5 ± 3 °C, 25 ± 2 °C/60 ± 5% relative humidity (RH), and 40 ± 2 °C/75 ± 5%RH) for 6 months. The stability of the optimized HVD-NLCs formulation was examined by measuring PS, PDI, ZP, and total drug content at 0, 1, 3, and 6 months.

#### Statistical Analysis

The results were analyzed using one-way analysis of variance (ANOVA) and followed by a post-hoc Tukey-HSD. The statistical analysis was performed using IBM® SPSS® Statistical software (Version 22, NY, USA). All the values were expressed as the mean and standard deviation (mean ± SD). The difference was statistically significant when *p* < 0.05.

## Results and Discussion

### Analysis Using 2^4^ Full Factorial Design

The values of the selected independent variables, i.e., homogenization time (A), solid lipid concentration (B), liquid lipid concentration (C), and surfactant concentration (D) and the obtained results of the selected responses, i.e., PS, PDI, ZP, and %EE are shown in Table 2. Each independent variable and their interactions were evaluated statistically using the ANOVA. The polynomial coefficient for each dependent variable was generated by the Design-Expert® software to quantify the effect of each independent variable, as shown in Eqs. 3, 4, 5, and 6. The equations only represented the significant factors and their interactions.

**Table 2:** Observed responses (mean) in 2^4^ full factorial design for HVD-NLCs

Formula code	Factors				Responses			
	*A*	*B*	*C*	*D*	PS (nm)	PDI	ZP (mV)	EE (%)
VER-1	20	270	15	200	207.33 ± 5.68	0.562 ± 0.019	− 37.5 ± 1.17	74.35 ± 4.00
VER-2	30	270	15	200	183.7 ± 5.38	0.629 ± 0.012	− 40.4 ± 1.31	61.01 ± 3.06
VER-3	20	285	15	200	204.5 ± 3.078	0.556 ± 0.049	− 43.8 ± 1.35	77.63 ± 9.36
VER-4	30	285	15	200	189.86 ± 3.76	0.546 ± 0.049	− 43.7 ± 1.31	83.15 ± 6.08
VER-5	20	270	30	200	203.06 ± 4.65	0.409 ± 0.052	− 45.9 ± 2.71	72.33 ± 9.64
VER-6	30	270	30	200	209.5 ± 3.47	0.327 ± 0.037	− 47.5 ± 2.45	72.08 ± 3.95
VER-7	20	285	30	200	201.46 ± 2.36	0.383 ± 0.029	− 49.1 ± 0.953	76.48 ± 17.81
VER-8	30	285	30	200	182.6 ± 6.95	0.382 ± 0.022	− 48.1 ± 1.36	67.11 ± 5.37
VER-9	20	270	15	300	188.4 ± 4.9	0.462 ± 0.043	− 49.1 ± 1.55	97.21 ± 1.615
VER-10	30	270	15	300	170.73 ± 5.53	0.526 ± 0.03	− 45.6 ± 1.17	91.71 ± 9.19
VER-11	20	285	15	300	190.3 ± 8.22	0.451 ± 0.033	− 46.7 ± 1.7	98.81 ± 1.012
VER-12	30	285	15	300	167.96 ± 1.76	0.589 ± 0.101	− 44.5 ± 3.5	84.56 ± 0.162
VER-13	20	270	30	300	173.46 ± 0.75	0.501 ± 0.019	− 46.7 ± 1.13	93.95 ± 4.10
VER-14	30	270	30	300	157.46 ± 3.61	0.429 ± 0.023	− 49.6 ± 2.39	79.86 ± 1.15
VER-15	20	285	30	300	178.36 ± 2.65	0.489 ± 0.048	− 48.1 ± 1.58	95.39 ± 2.16
VER-16	30	285	30	300	162.86 ± 3.35	0.436 ± 0.031	− 49.4 ± 1.08	77.88 ± 2.78

*A* homogenization time (min), *B* solid lipid concentration (mg), *C* liquid lipid concentration (mg), *D* surfactant concentration (mg), *PS* particle size, *PDI* polydispersity index, *ZP* zeta potential, *EE* entrapment efficiency

3 $$ \mathrm{PS}=185.73-7.64A-2.13C-12.03D-2.15\mathrm{AC}+2.16\mathrm{BD}-3.53\mathrm{CD} $$

4 $$ \mathrm{PDI}=0.48-0.060C-0.029\mathrm{AC}+0.039\mathrm{CD} $$

5 $$ \mathrm{ZP}=-45.94-2.06C-1.41D+0.91\mathrm{BD}-1.09\mathrm{CD} $$

6 $$ \%\mathrm{EE}=81.40-4.31A+8.45D-2.13\mathrm{AD}+1.90\mathrm{BD} $$

where *A* is the homogenization time (min), *B* is the solid lipid concentration (mg), *C* is the liquid lipid concentration (mg), and *D* is the surfactant concentration (mg). The positive and negative signs before each factor or interaction factors in the equations are representing either a synergistic or an antagonistic effect, respectively. The response surface plots were generated to observe the concurrent effect of each factor or factor interactions on the response parameters.

### Effects of Variables on Mean Particle Size

As shown in Eq. 3, factors *A*, *C*, and *D* have antagonistic effects on particle size (PS). It could be concluded that increasing the homogenization time (factor *A*) is simultaneously associated with a significant decrease in the PS of NLCs (*p* < 0.05). This is probably due to the high shear force of homogenization that efficiently breaks the lipid globules into smaller particles. Similarly, increasing the liquid lipid concentration (*C*) was found to concurrently decrease the PS of the NLCs. This might be attributed to the ability of oleic acid to decrease the viscosity of the system as well as the surface tension when combined with Compritol 888 ATO®, hence produced smaller particle size of NLCs. The ability of the liquid lipid to decrease the viscosity of the system when combined with the solid lipid was also reported by Jenning et al. when they prepared a SLN dispersion [5]. The authors observed that increasing the liquid lipid concentration from 0 to 38% in the lipid matrix composition would reduce the particle size. Furthermore, increasing the surfactant concentration (*D*) would decrease the PS, because higher amount of surfactant present in the system would easily emulsifies the total lipid contents in the formulation, thus forms smaller nanoparticles.

**Fig. 2:**
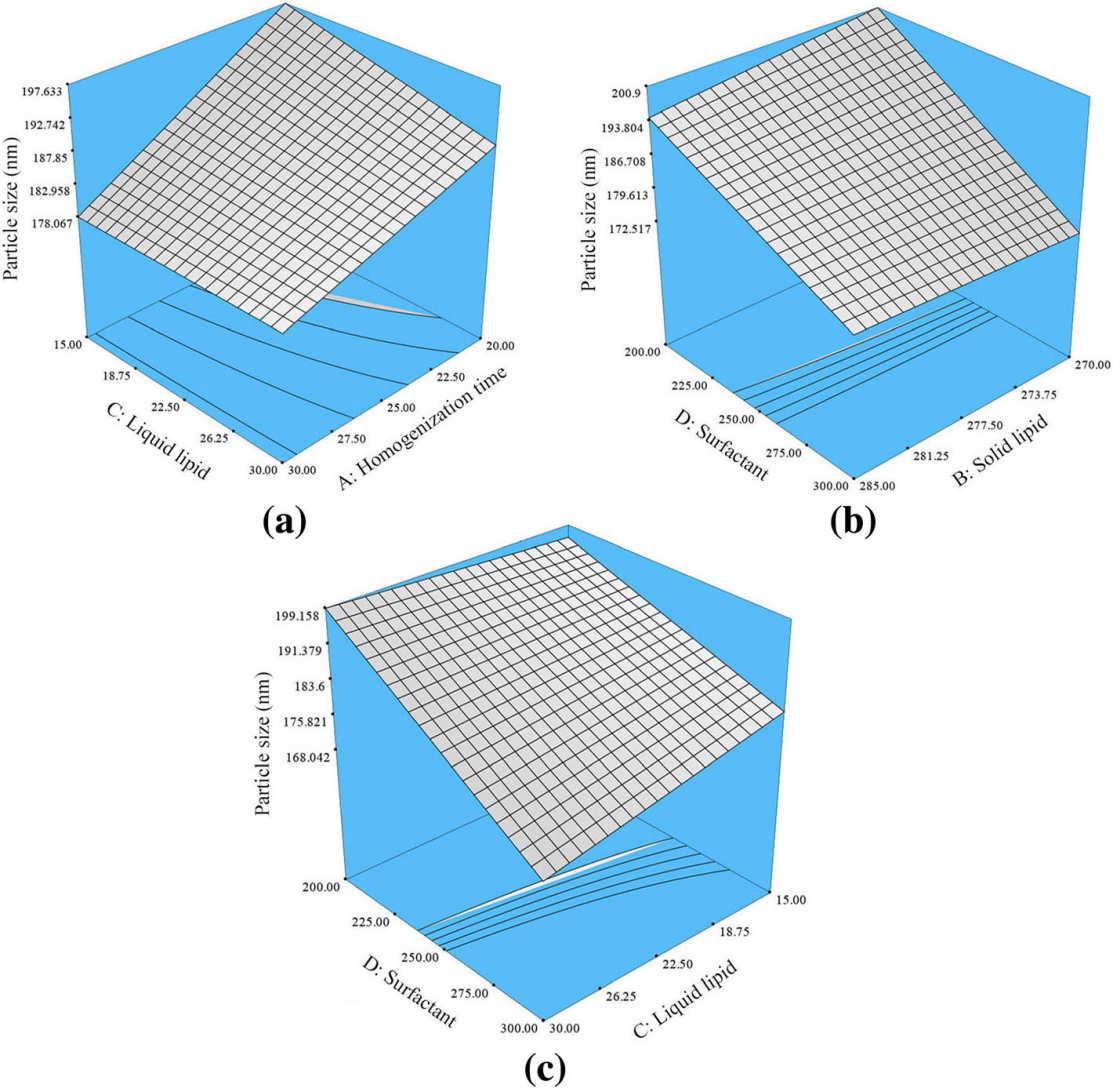
Response surface plot depicting the significance (*p* < 0.05) effect of homogenization time, liquid lipid concentration, surfactant concentration, and interaction between **a** AC, **b** BD, and **c** CD on the PS of HVD-NLCs. *A* homogenization time, *B* solid lipid, *C* liquid lipid, *D* surfactant

Figure 2 shows the significant (*p* < 0.05) effect of factors (AC, BD, and CD) interactions on the PS of the NLC. The positive impact of factor (BD), and the negative impact of factors (AC and CD interaction) showed a significant effect on the NLCs particle size. The negative impact of factor (AC) showed that at longer homogenization time and higher liquid lipid concentration caused a decrease in PS. The interaction between higher concentrations of solid lipid and surfactant in the NLC formulation (BD interaction) showed a significant positive impact on the particle size, which resulted in larger particle size. In contrast, the interaction between higher concentrations of the liquid lipid and surfactant (CD interaction) showed a significant negative impact on the particle size where smaller size was produced.

### Effects of Variables on PDI

**Fig. 3:**
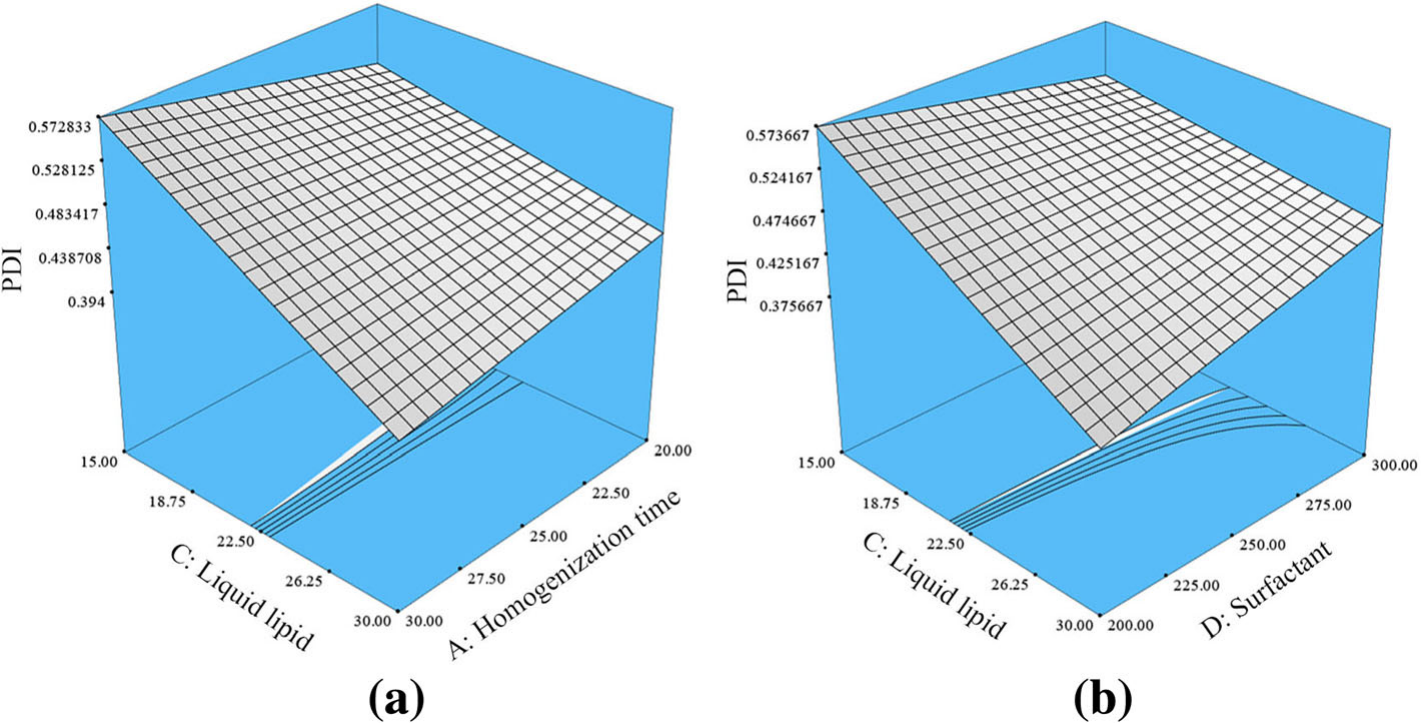
Response surface plot depicting the significant (*p* < 0.05) effect of liquid lipid concentration and interactions between **a** AC and **b** CD on PDI of HVD-NLCs. *A* homogenization time, *C* liquid lipid, *D* surfactant

As illustrated in Eq. 4, oleic acid is shown to have a negative impact on the PDI, which means that increasing oleic acid concentration would lower PDI values and lead to better distribution of the NLC particles. Similar effect of oleic acid on NLC was also observed by some researches [6]. Figure 3 depicted the interaction between the homogenization time and the liquid lipid concentration (AC interaction). This interaction showed a significant negative effect on the PDI. It means that longer homogenization time and higher liquid lipid concentration would lead to a decrease in the PDI values. In addition, the interaction between the liquid lipid and surfactant concentrations (CD interaction) showed a synergistic effect on the PDI values. Thus, simultaneously increasing the liquid lipid and surfactant concentrations would increase the PDI values and produce less homogenous HVD-NLCs particles. This might be due to further increase in the surfactant concentration beyond the optimized value may cause an accumulation of the surfactant at the outer surfaces of nanoparticles, which in turn increases the PDI values. The same pattern was also observed by Shamma et al., whereby increasing the surfactant concentration caused an increase in PDI values of NLCs [7].

### Effects of Variables on ZP

The HVD-NLCs formulations had the negative charge due to the ionization of glyceryl behenate (a fatty acid in Compritol 888 ATO®), oleic acid, and dextran sulfate residue. Based on Eq. 5, increasing the amount of oleic acid had the negative impact on the nanoparticles, and resulting in increase in the negative charge of the nanoparticles, hence increase ZP of HVD-NLCs. This is possibly due to excessive ionization of abundant carboxylic groups in the oleic acid (liquid lipid) [8]. Moreover, glyceryl behenate (solid lipid) also showed a negative effect on the ZP values, where increasing its concentration increases the ZP of nanoparticles. This is due to higher ionization of carboxylic in the glyceryl behenate that lead to higher negative charges at the outer surface of the nanoparticles.

**Fig. 4:**
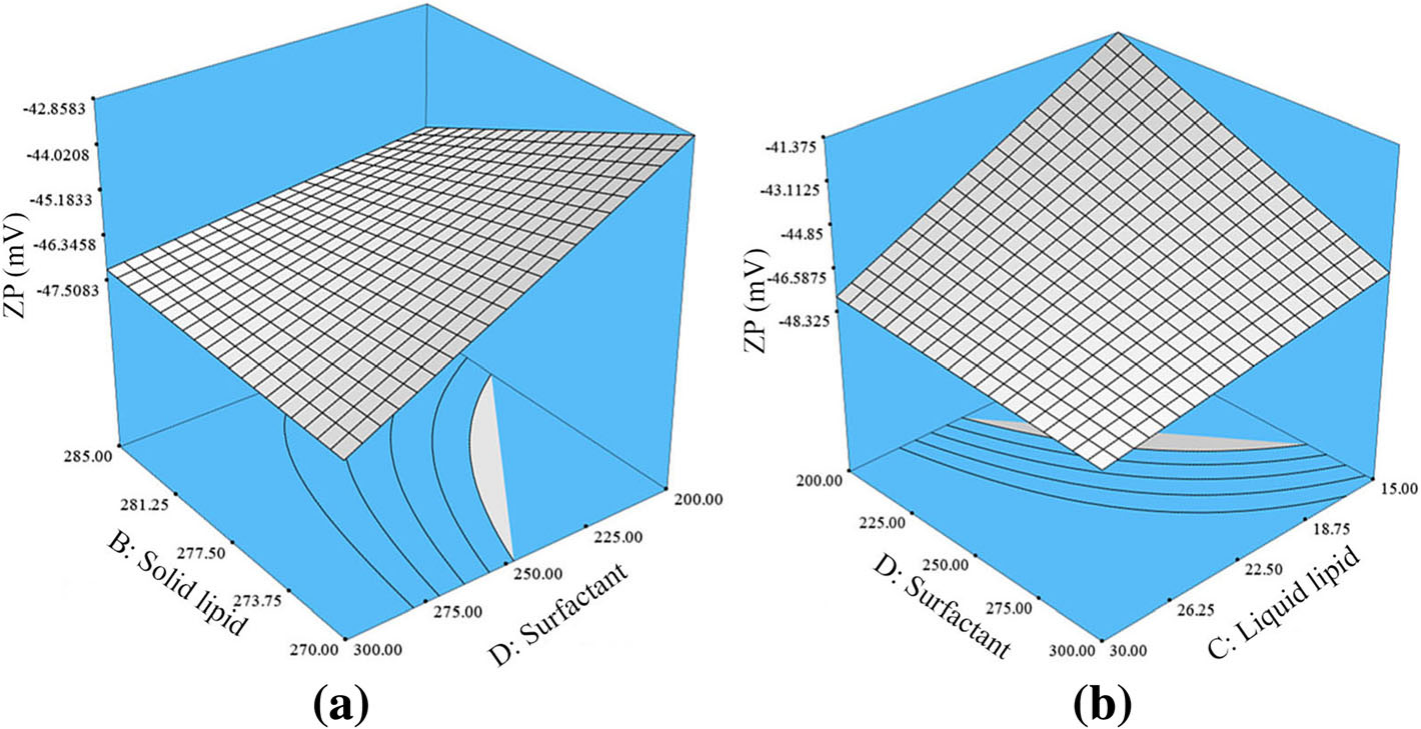
Response surface plot depicting the significant (*p* < 0.05) effect of liquid lipid concentration, surfactant concentration, and interactions between **a** BD and **b** CD on the ZP of HVD-NLCs. *B* solid lipid, *C* liquid lipid, *D* surfactant

The BD interaction had a significant positive effect on the ZP of NLCs, while CD interaction showed a negative effect (Fig. 4). Increasing the concentration of solid lipid and decreasing the concentration of surfactant reduces the negative charge of the nanoparticles and decreases the ZP values. Probably, at this stage, lower surfactant concentration would not be able to ionize the total amount of solid lipid, that lead to reduce the negative charges on the nanoparticles surface, thus reduce the ZP of HVD-NLCs. Furthermore, simultaneously increasing the concentration of liquid lipid and surfactant (CD interaction) would significantly increase the ZP value of HVD-NLCs. This is because more carboxylic acid would be ionized in the presence of higher surfactant concentration in the formulation.

### Effects of Variables on %EE

Equation 6 showed that increasing the homogenization time would significantly (*p* < 0.05) decrease the %EE of the drug. Longer homogenization time could strip or peel off the drug molecules attached at the outer surface of the nanoparticles, which resulted in a lower %EE of the drug in the HVD-NLCs. In contrast, increasing the surfactant concentration resulted in a higher %EE of the drug in the HVD-NLCs. This could be due to higher surfactant concentration would increase its outer surface coating of NLCs, where part of the drug would be entrapped in it. Zhuang et al. and Das et al. suggested that an adequate concentration of surfactant is essential for the solubilization of drug molecules inside the lipid lattice and at the outer surface of nanoparticles [9, 10].

**Fig. 5:**
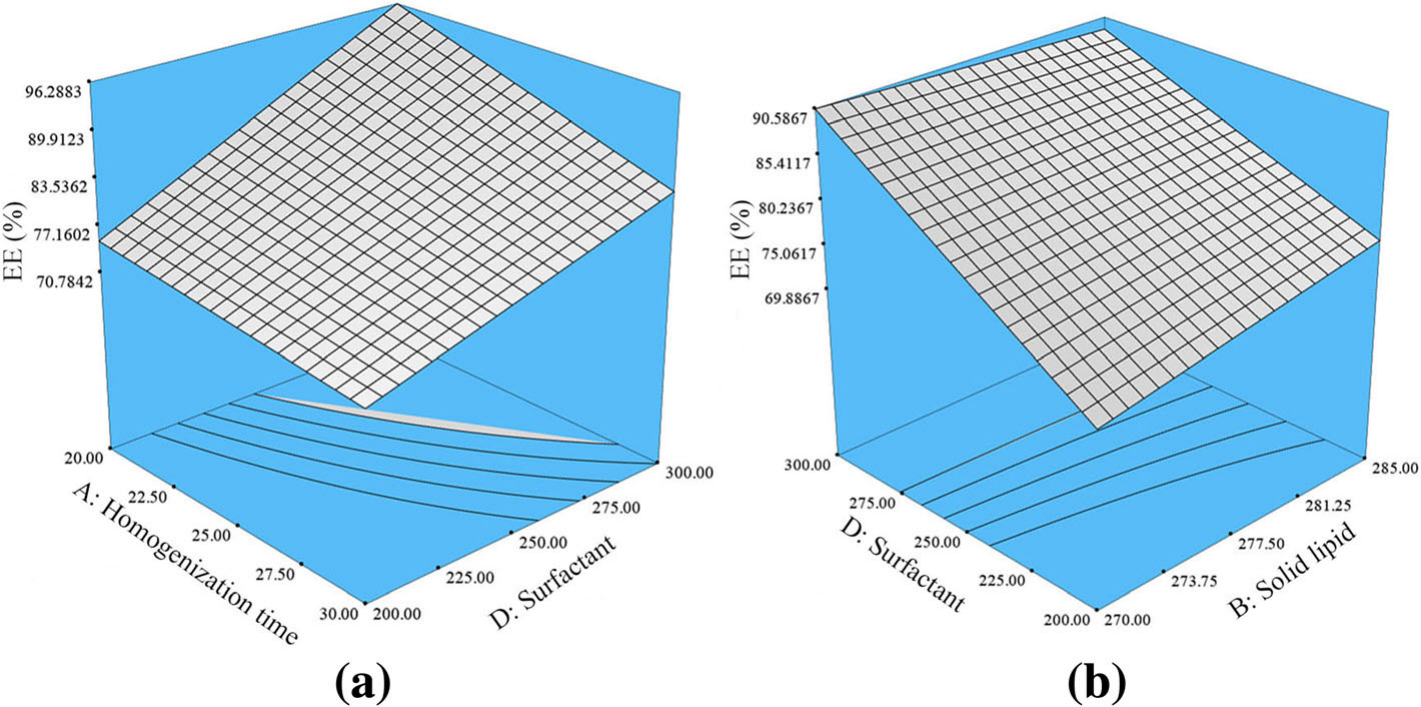
Response surface plot depicting the significant (*p* < 0.05) effect of homogenization time, liquid lipid concentration, surfactant concentration, and interactions between **a** AD and **b** BD on EE of verapamil in HVD-NLCs. *A* homogenization time, *B* solid lipid, *D* surfactant

As shown in Fig. 5, AD interaction had a significant (*p* < 0.05) negative impact, whereby increasing the homogenization time and decreasing the amount of surfactant lowers the %EE of drug in the HVD-NLCs. In contrast, the interaction between solid lipid and surfactant concentrations (BD interaction) showed a positive effect on %EE of drug. Therefore, increasing the concentration of solid lipid and surfactant will increase the %EE of the drug in the HVD-NLCs. This is probably due to the higher surfactant concentration which would solubilize the excess drug molecules, and at the same time higher amount of solid lipid presence in the system has accommodated the excess drug molecules, hence increase the %EE of the drug in the HVD-NLCs.

### Selection of Optimized HVD-NLCs Using the Desirability Function

**Fig. 6:**
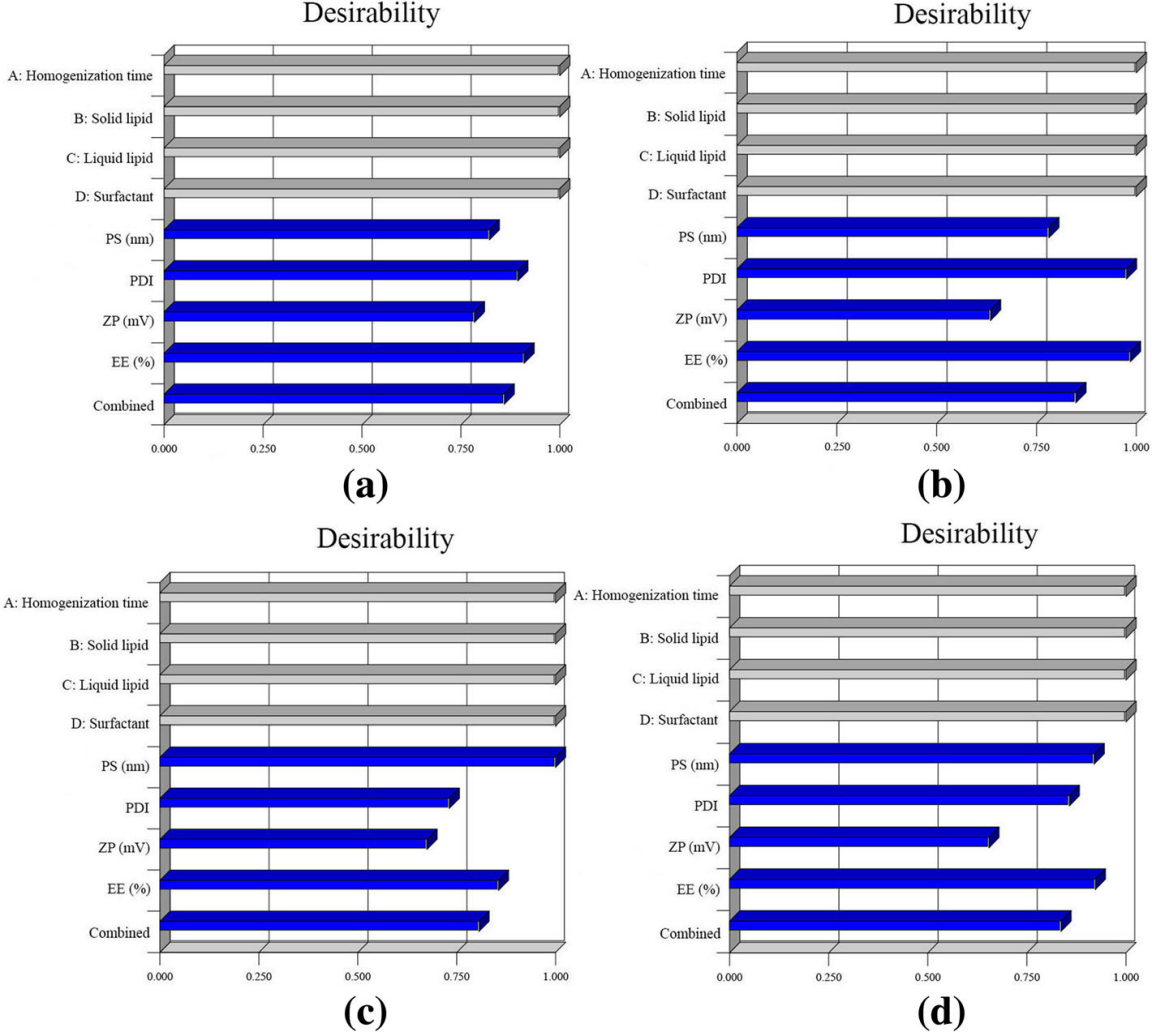
Bar graph displaying the individual and combined desirability of several responses on selected HVD-NLCs formulations. **a** VER-9, **b** VER-11, **c** VER-13, and **d** VER-15

The HVD-NLCs formulations with the code no.VER-9, VER-11, VER-13, and VER-15 had higher desirability values of 0.863, 0.852, 0.811, and 0.840, respectively. These formulations were selected for further study because the desirability values were closer to 1. The individual and combined desirability function of all the responses for the selected formulations is depicted in Fig. 6. The mean PS, PDI, ZP, and %EE of these formulations were in the range of 173.46 ± 0.757 to 190.3 ± 8.22, 0.451 ± 0.033 to 0.501 ± 0.019, − 46.73 ± 1.13 to − 49.16 ± 1.55, and 93.95 ± 4.10 to 98.81 ± 1.01, respectively.

### Lyophilization Study

Lyophilization is one of the commonly used methods in the pharmaceutical field to change aqueous formulation into the dried form to prolong the physical and chemical stability of the nanoparticle during storage [11, 12]. However, the lyophilization step produces various stresses to the sample during the process. So, the need of cryoprotectant is essential to avoid the stress condition and protect the sample. Among the screened cryoprotectants, trehalose was found to be the most suitable one for protecting the HVD-NLCs formulations from aggregation after the lyophilization process. The HVD-NLCs formulations protected with trehalose had the smallest particle size and lowest PDI (Table 3). Trehalose offers many advantages over other cryoprotectants including less chemical interactions and exhibit higher glass transition temperature which may help to prolong the stability of nanoparticles. Furthermore, trehalose has been demonstrated as an efficient cryoprotectant for freeze drying of Compritol 888 ATO® (Glyceryl behenate) in the lipid-based nanoparticles [13, 14]. Thus, in the present study, trehalose was selected as cryoprotectant for the HVD-NLCs formulations.

**Table 3:** Screening of different cryoprotectants on the basis of mean PS and PDI

Cryoprotectants	(Lipid:cryoprotectant) ratio	Before lyophilization		After lyophilization	
		PS (nm)	PDI	PS (nm)	PDI
Mannitol	1:1	180.56 ± 3.53	0.575 ± 0.022	9153.57 ± 10,360.71	1 ± 0.00
Fructose	1:1			481.42 ± 41.78	0.941 ± 0.102
Sucrose	1:1			532.98 ± 231.80	1 ± 0.00
Trehalose	1:1			214.92 ± 3.75	0.554 ± 0.039
Lactose	1:1			2153.92 ± 1830.25	1 ± 0.00

*PS* particle size, *PDI* polydispersity index

In the next study, different ratios of trehalose were used in the HVD-NLCs formulations after or during the homogenization process. Table 4 shows the effects of different ratios of trehalose added into the formulations after homogenization on PS, PDI, and %EE.

**Table 4:** Addition of trehalose after homogenization

Formula code	(Lipid:trehalose) ratio	Testing condition	PS (nm)	PDI	% EE
VER-9	1:0	Before lyophilization	178.34 ± 4.85	0.534 ± 0.0002	97.98 ± 0.96
		After lyophilization	372.78 ± 10.81	0.733 ± 0.048	97.61 ± 0.51
	1:1	After lyophilization	212.82 ± 3.83	0.544 ± 0.044	67.47 ± 1.61
	1:2		329.45 ± 12.10	0.995 ± 0.007	40.68 ± 7.32
	1:4		227.93 ± 3.64	0.634 ± 0.812	22.19 ± 3.25
	1:6		317.94 ± 65.10	0.812 ± 0.052	9.47 ± 2.93
	1:8		260.67 ± 16.35	0.611 ± 0.033	8.37 ± 2.02
VER-11	1:0	Before lyophilization	166.34 ± 3.84	0.465 ± 0.012	99.09 ± 0.29
		After lyophilization	1544.18 ± 2064	1 ± 0.00	98.27 ± 0.34
	1:1	After lyophilization	171.59 ± 3.69	0.558 ± 0.033	70.28 ± 0.87
	1:2		210.97 ± 48.38	0.610 ± 0.041	44.88 ± 3.55
	1:4		264.33 ± 13.52	0.748 ± 0.022	23.81 ± 3.17
	1:6		231.9 ± 17.64	0.549 ± 0.025	9.06 ± 2.75
	1:8		260.33 ± 16.30	0.506 ± 0.078	7.96 ± 2.56
VER-13	1:0	Before lyophilization	165.5 ± 4.34	0.524 ± 0.174	93.90 ± 0.77
		After lyophilization	1129.41 ± 657.64	1 ± 0.00	92.77 ± 1.37
	1:1	After lyophilization	261.11 ± 13.35	0.608 ± 0.025	71.53 ± 2.85
	1:2		188.66 ± 9.19	0.662 ± 0.044	52.81 ± 2.50
	1:4		256.45 ± 22.60	1 ± 0.00	25.19 ± 3.90
	1:6		272.61 ± 34.13	0.622 ± 0.025	10.64 ± 0.82
	1:8		238.02 ± 12.73	0.824 ± 0.030	8.43 ± 3.08
VER-15	1:0	Before lyophilization	174.52 ± 2.97	0.497 ± 0.012	95.87 ± 0.25
		After lyophilization	1175.55 ± 1016.11	1 ± 0.00	94.90 ± 1.26
	1:1	After lyophilization	232.77 ± 4.55	0.497 ± 0.069	74.95 ± 3.18
	1:2		300.91 ± 21.33	0.737 ± 0.036	46.04 ± 9.01
	1:4		270.42 ± 19.17	0.659 ± 0.094	27.28 ± 4.32
	1:6		289.23 ± 42.33	0.824 ± 0.030	9.39 ± 2.48
	1:8		208.55 ± 10.87	0.738 ± 0.022	7.78 ± 3.00

*PS* particle size, *PDI* polydispersity index, *EE* entrapment efficiency

The mean PS was found in the nano size range, while PDI was < 1 in all ratios of lipids to trehalose used in the study. However, the %EE of verapamil was decreased significantly with increasing the ratio of trehalose to lipids. The %EE of lipid:trehalose at the ratio of 1:1 was significantly higher than the lipid:trehalose ratio of > 1:1. Increasing the amount of trehalose beyond the lipid trehalose ratio of 1:1 enhanced the removal of verapamil from the verapamil-dextran sulfate complex. This is possibly due to the interaction between trehalose and dextran at the outer surface of nanoparticles, which promotes the displacement of verapamil from the verapamil-dextran sulfate complex and hence, lowers the %EE of drug in the HVD-NLCs. The interaction between trehalose and dextran takes place due to the formation of sugar-salt complex (trehalose-dextran complex), in that the trehalose (sugar) competes with cationic verapamil and forms complex with polyanionic dextran [15]. Miller et al. measured the viscosity and glass transition temperature of trehalose with boric acid and proposed the formation of chemical complex between trehalose and borate ion (B(OH)_4_^−^) [15]. Therefore, based on the obtained results, the lipid to trehalose ratios of 1:1 and 1:2 were selected for the next study of trehalose addition during homogenization. Table 5 shows that the results of PS, PDI, and %EE were significantly better when trehalose were added during homogenization. This could be due to better mixing of trehalose in the HVD-NLCs formulation by the homogenization process. It is evident from the data that there was no significant difference in the %EE of lipid:trehalose ratios of 1:1 and 1:2. Therefore, formulation VER-9 and VER-11 at lipid:trehalose ratio of 1:1 was selected for further studies.

**Table 5:** Addition of trehalose during homogenization (after lyophilization)

Formula code	(Lipid:trehalose) ratio	PS (nm)	PDI	% EE
VER-9	1:1	192.29 ± 2.98	0.553 ± 0.075	94.06 ± 2.55
VER-9	1:2	184 ± 3.84	0.694 ± 0.076	93.26 ± 2.66
VER-11	1:1	195.78 ± 4.58	0.568 ± 0.029	94.25 ± 1.57
VER-11	1:2	211.72 ± 3.13	0.760 ± 0.108	92.17 ± 1.67

*PS* particle size, *PDI* polydispersity index, *EE* entrapment efficiency

### In Vitro Release of HVD-NLCs

**Fig. 7:**
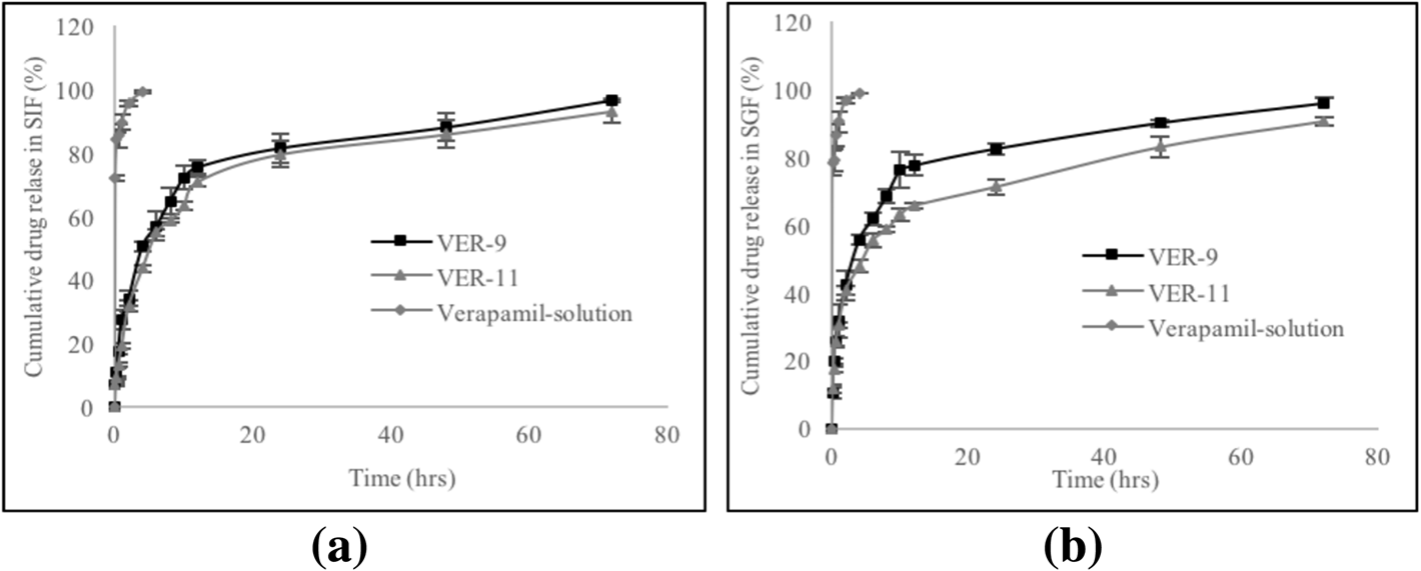
Cumulative percentage of verapamil released in **a** SIF (pH 6.8) and **b** SGF (pH 1.2) release media for 72 h

The release profile of verapamil solution from the selected HVD-NLCs formulations (VER-9 and VER-11) in SGF and SIF are shown in Fig. 7. The release profile of verapamil from the VER-9 and VER-11 formulations was significantly longer than verapamil solution in both SGF and SIF media. Verapamil was loaded as an ionic complex with dextran sulfate in the HVD-NLCs formulation. This could be the reason for the sustained release of verapamil from the HVD-NLCS. The formulations released about ~ 85% of verapamil after 48 h and the drug had a prolonged release profile up to 72 h. On the other hand, the verapamil solution released approximately 99% of verapamil within 4 h. The release profiles of the selected formulations VER-9 and VER-11 were found almost similar in SGF and SIF release media. However, the release of verapamil from VER-9 was found slightly better as compared to VER-11 in the SGF release medium. The percentage cumulative release of verapamil at 72 h from VER-9 (95.91 ± 1.40) was found significantly higher as compared to VER-11 (90.67 ± 1.16) in the SGF medium. Therefore, VER-9 was selected as a final formulation and proceeded for further investigations.

### DSC Study

**Fig. 8:**
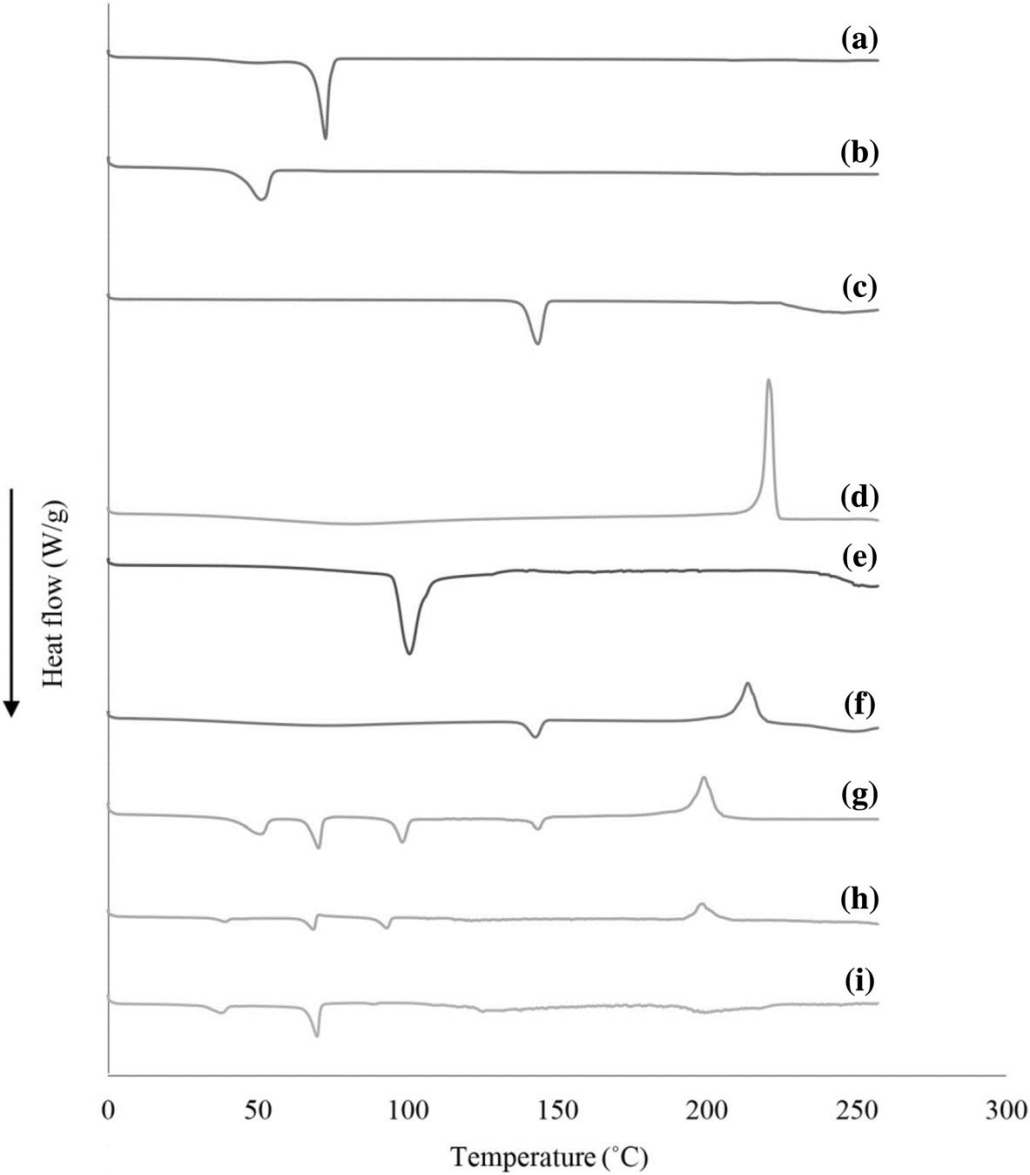
DSC thermograms of **a** Compritol 888 ATO, **b** Polomxamer 188, **c** verapamil HCl, **d** dextran sulfate, **e** trehalose, **f** verapamil HCl and dextran sulfate physical mixture, **g** physical mixture (including Compritol 888 ATO, poloxamer 188, trehalose, verapamil, and dextran sulfate), **h** placebo, and **i** HVD-NLCs formulation (VER-9)

The DSC analysis of verapamil, dextran sulfate, Compritol 888 ATO®, poloxamer 188, trehalose, physical mixture (including Compritol 888 ATO®, poloxamer 188, trehalose, verapamil, and dextran sulfate), lyophilized blank formulation (Placebo), and lyophilized HVD-NLCs formulation (VER-9) are shown in Fig. 8. Verapamil, dextran sulfate, Compritol 888 ATO®, poloxamer 188, and trehalose had the melting peak at 144.96 °C, 220.41 °C, 72.46 °C, 50.80 °C, and 100.13 °C, respectively. The thermograms showed that there was no significant shift in the peaks of physical mixture, lyophilized blank formulation, and HVD-NLCs formulation (VER-9) when compared to the individual peak components such as Compritol 888 ATO®, poloxamer 188, trehalose, verapamil, and dextran sulfate. This indicated the physical compatibility between the components. The endothermic peak of verapamil at 144.96 °C was no longer seen in the thermogram of HVD-NLCs formulation (VER-9) (Fig. 8f), which could be due to verapamil that had changed to amorphous form when incorporated in the HVD-NLCs matrix [16].

### WAXS Study

**Fig. 9:**
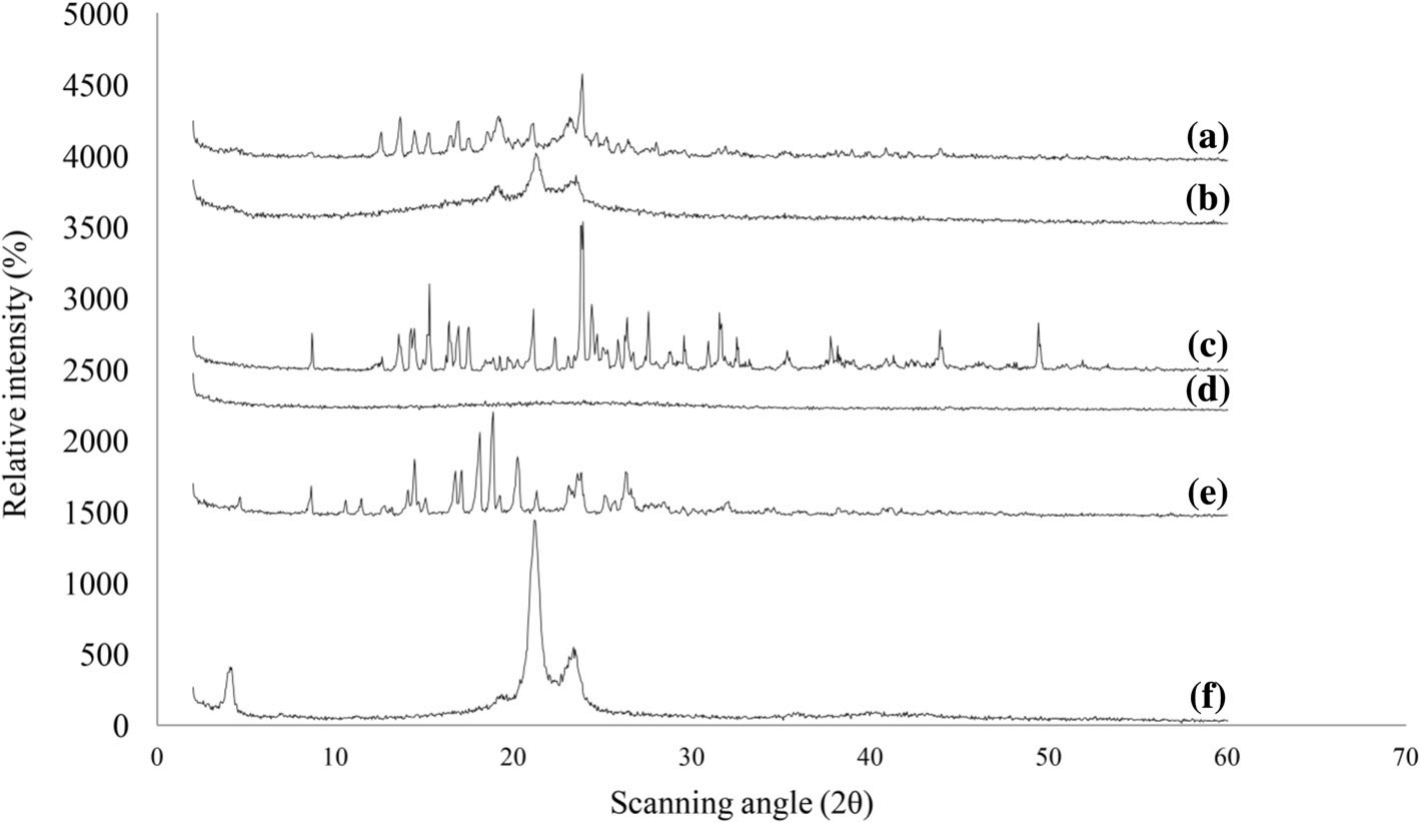
X-ray patterns of **a** lyophilized HVD-NLCs formulation (VER-9), **b** lyophilized blank formulation, **c** trehalose, **d** dextran sulfate, **e** verapamil HCl, and **f** Compritol 888 ATO

The WAXS/2θ scans of pure verapamil, dextran sulfate, bulk Compritol 888 ATO®, trehalose, lyophilized blank formulation (containing Compritol 888 ATO®, oleic acid, poloxamer 188, Tween 80®, trehalose, and dextran sulfate), and lyophilized HVD-NLCs formulation (VER-9) are shown in Fig. 9. The diffraction peaks of pure verapamil showed specific crystalline nature of the drug at 2θ scattered angles 14.42°, 16.67°, 17.07°, 18.07°, 18.82°, 20.22°, 23.77°, and 26.27° [17]. However, the WAXS/2θ scans of HVD-NLCs formulation (VER-9) showed that the peaks of verapamil diminished indicating a decrease in the crystallinity of the drug when incorporated in the lipids of HVD-NLCs. The scan also showed the predominant peaks at 2θ scattered angles 12.47°, 15.17°, 16.87°, 21.07°, and 23.82° which possibly attributed to the crystalline structure of Compritol 888 ATO® and trehalose because none of these peaks showed similarity with any of the pure verapamil peaks [10]. The results suggested that verapamil was in the amorphous form when incorporated in the HVD-NLCs formulation. The Compritol 888 ATO® revealed polymorphic crystalline transformation upon heating, whereby it changed to a more stable form, and showed reduced intensity peaks at 21.1° and 23.3° in the HVD-NLCs and blank formulations. The WAXS pattern of pure dextran sulfate indicated its amorphous form.

### Electron Microscopic Examinations

**Fig. 10:**
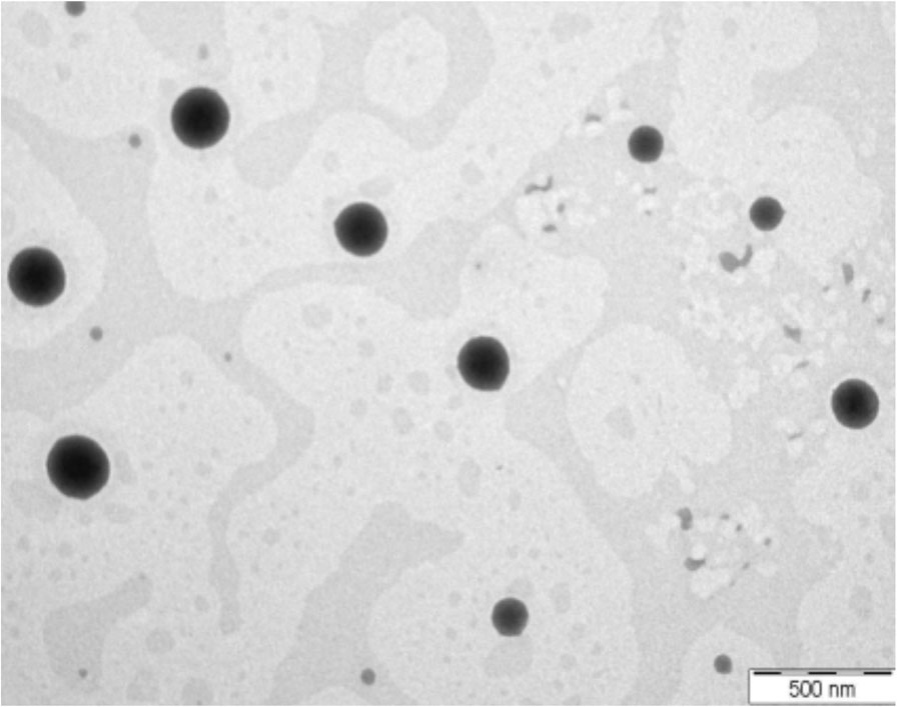
TEM image of VER-9 before lyophilization

**Fig. 11:**
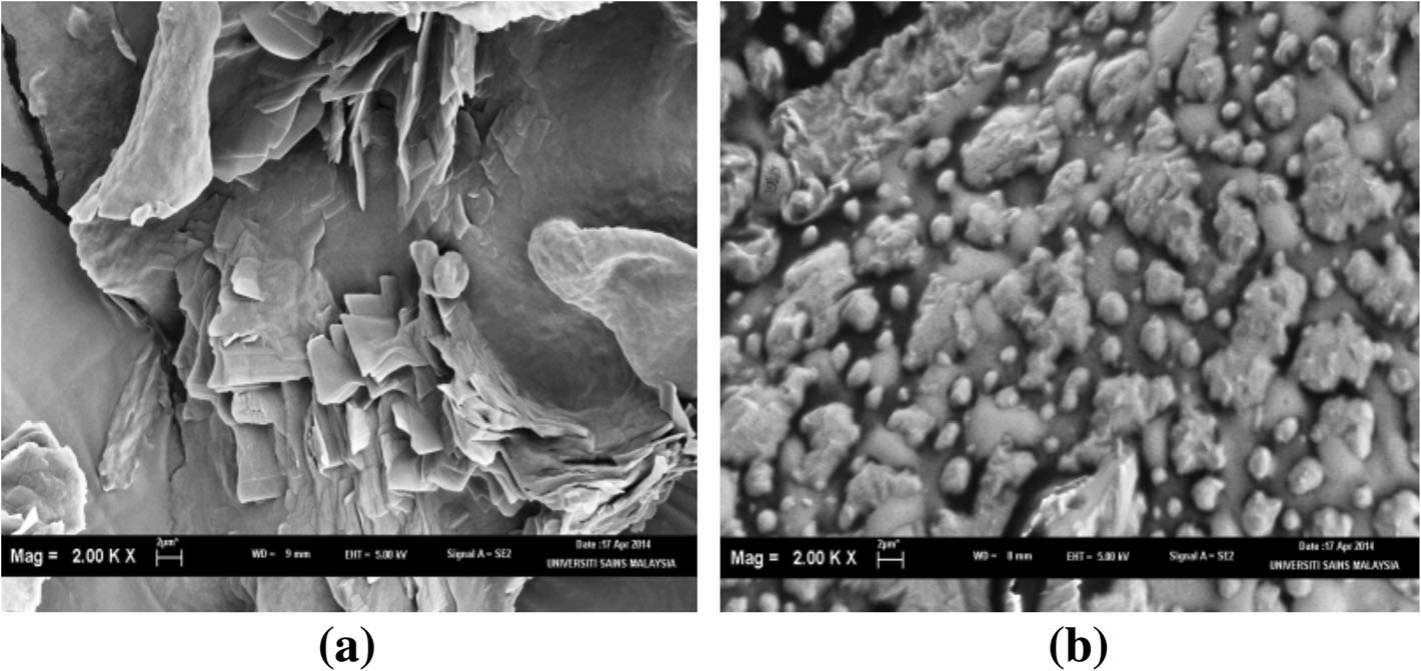
SEM images of VER-9 formulation **a** without trehalose; **b** with trehalose

The particle shape and size of the HVD-NLCs formulation (VER-9) were examined using the electron microscope. The liquid preparation of HVD-NLCs formulation (VER-9) observed under transmission electron microscope (TEM) showed that the particles had a spherical shape with the size less than 200 nm (Fig. 10). In contrast, observation under scanning electron microscope (SEM) revealed that the nanoparticles no longer had a spherical shape following lyophilization of the formulation without trehalose. However, the cryoprotective effect of trehalose could be seen when it was incorporated in the HVD-NLCs formulation (VER-9). The SEM image indicated that the trehalose helped to protect the structure of the nanoparticle to such extent when compared to the lyophilized formulation without the cryoprotectant (Fig. 11).

### Cellular Uptake Study of HVD-NLCs

**Fig. 12:**
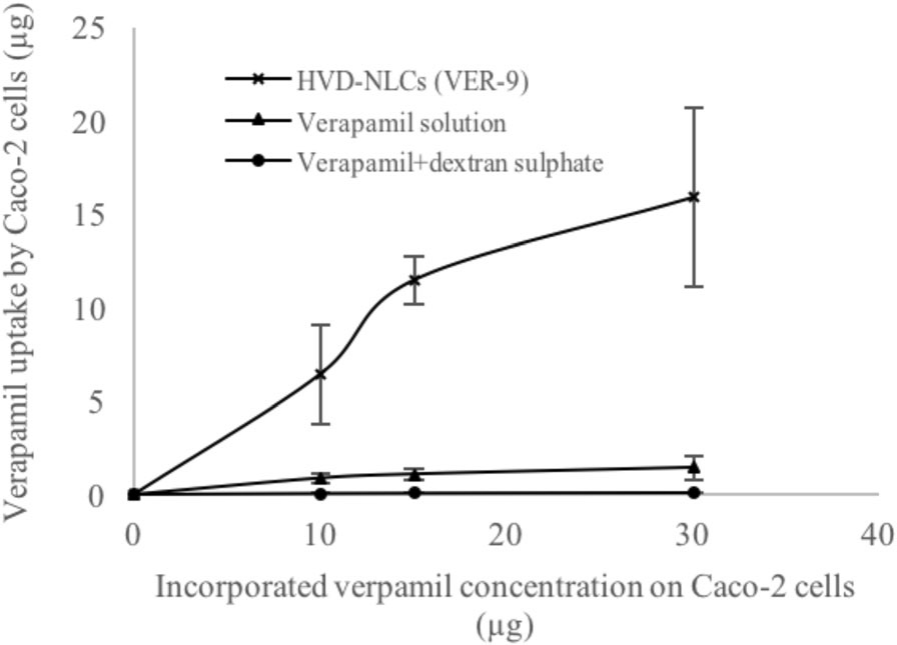
In vitro model of Caco-2 cell line showing cellular uptake of HVD-NLCs (VER-9), verapamil solution, and verapamil-dextran complex (mean ± SD, *n* = 3)

Caco-2 cells have been extensively used to study drugs intestinal cellular uptake [18]. The Caco-2 cells lines is spontaneously differentiating to form monolayer which has significant morphological and biological resemblance to the intestinal epithelium [19]. Several researchers have reported the in vitro cytotoxicity studies of Compritol ATO 888® [20], oleic acid [21, 22], Tween 80® [23, 24], and poloxamer 188 [25]. These substances were used as excipients in the optimized formulation of HVD-NLCs (VER-9). Based on their finding, these excipients were safe within the concentration used, the cell line type, and cell density that have been employed in the present study. The concentration of verapamil in the samples of VER-9, verapamil solution, and verapamil-dextran complex used in the cellular uptake study was in the range of 10.03 μg/200 μL to 30.1 μg/200 μL. It was found that the verapamil cellular uptake was increased with increasing the verapamil concentration in the samples. The cellular uptake values of verapamil from VER-9, verapamil solution, and verapamil-dextran complex were increased from 6.45 ± 2.62 to 15.92 ± 4.78 μg, 0.89 ± 0.28 to 1.46 ± 0.65 μg, and 0.064 ± 0.019 to 0.118 ± 0.003 μg, respectively (Fig. 12). The verapamil cellular uptake from the VER-9 formulation was 10.90- and 134.91-fold higher than the verapamil solution and verapamil-dextran complex, respectively. The results indicated that verapamil was passively transported from HVD-NLCs, verapamil solution, and verapamil-dextran complex in the caco2-cells. A similar finding of higher cellular uptake of drugs from a lipid-based nanoformulation was reported [26].

Caco-2 cells act similarly as cell in animals when stimulated by fatty acid. van Greevenbroek et al. suggested that the incorporation of long-chain unsaturated fatty acids (e.g., oleic acid and linoleic acid) into the Caco-2 cells stimulate the secretion of very low-density lipoproteins (VLDL) and chylomicrons. In contrast, administration of saturated fatty acids primarily secretes intermediate- and low-density lipoproteins (IDL and LDL) [27]. Similar findings of increasing chylomicron secretion by the long-chain fatty acids were also reported by Caliph et al. in animal model [28]. In the present investigation, the long-chain fatty acids (Compritol 888 ATO® and oleic acid) used in the preparation of the HVD-NLCs may also stimulate the secretion of chylomicron, and hence increase the cellular uptake of the model drug.

### Stability Study

The lyophilized VER-9 formulation was found stable for 6 months at refrigerated condition (5 ± 3 °C) (Table 6). There were no significant differences in the PS, PDI, ZP, and %EE after 6 months of stability study at storage temperature of 5 ± 3 °C. However, the VER-9 formulation was not stable after 1-month storage at the temperatures of 25 ± 2 °C/60 ± 5%RH and 40 ± 2 °C/75 ± 5% RH. The PS and PDI were increased significantly, while the total drug content decreased significantly. After 1 month of stability study, the samples were not measurable due to aggregation and sticky behavior of the nanoparticles.

**Table 6:** Stability profile of lyophilized HVD-NLCs (VER-9) in terms of PS, PDI, ZP, and drug content (*n* = 3) in different storage conditions

Stability parameters	0 month	1 month	3 month	6 month
Storage condition (5 ± 3 °C)				
PS (nm)	193.76 ± 2.10	191 ± 3.17	192.46 ± 4.36	198.43 ± 2.90
PDI	0.542 ± 0.075	0.556 ± 0.052	0.554 ± 0.321	0.525 ± 0.251
ZP (mV)	− 48.5 ± 1.31	− 45.3 ± 2.55	− 42 ± 2.65	− 46.11 ± 3.11
Drug content (%EE)	100.03 ± 0.03	99.53 ± 0.72	99.06 ± 1.23	98.46 ± 1.67
Storage condition (25 ± 2 °C/60 ± 5%RH)				
PS (nm)	193.76 ± 2.10	962.16 ± 12.36	NM	NM
PDI	0.542 ± 0.075	1	NM	NM
ZP (mV)	− 48.5 ± 1.31	− 46.25 ± 2.36	NM	NM
Drug content (%EE)	100.03 ± 0.03	92.69 ± 0.75	87.56 ± 1.26	82.14 ± 1.90
Storage condition (40 ± 2 °C/75 ± 5% RH)				
PS (nm)	193.76 ± 2.10	865.12 ± 15.26	NM	NM
PDI	0.542 ± 0.075	0.963 ± 0.056	NM	NM
ZP (mV)	− 48.5 ± 1.31	− 49.47 ± 2.45	NM	NM
Drug content (%EE)	100.03 ± 0.03	91.78 ± 0.44	68.24 ± 1.80	65.84 ± 7.88

*NM* not measurable, *PS* particle size, *PDI* polydispersity index, *ZP* Zeta potential

## Conclusion

The lyophilized HVD-NLCs formulation was successfully developed and optimized. The lipid carriers had prolonged the release of verapamil from the HVD-NLCs formulation. The formulation was in the nano size range and stable for 6 months at 5 ± 3 °C. The cellular uptake of verapamil lipid formulation (NLCs) was found higher than the non-lipid formulations. This suggested that the NLCs as a lipid carrier for verapamil may play an important role in improving the absorption of the drug.

## Abbreviations

%EE: Percentage of entrapment efficiency
DMEM: Dulbecco’s modified Eagles medium
DSC: Differential scanning calorimetry
FBS: Fetal bovine serum
HVD-NLCs: Hybrid verapamil-dextran nanostructured lipid carriers
ICH: The international council for harmonization of technical requirements for pharmaceuticals for human
IDL: Intermediate density lipoproteins
LDL: Low density lipoproteins
NLCs: Nanostructured lipid carriers
PBS: Phosphate-buffered saline
PDI: Polydispersity index
PS: Particle size
RH: Relative humidity
SEM: Scanning electron microscope
TEM: Transmission electron microscope
VLDL: Very low-density lipoproteins
WAXS: Wide-angle X-ray scattering
ZP: Zeta potential
